# Host-Pathogen Interactions Mediated by MDR Transporters in Fungi: As Pleiotropic as it Gets!

**DOI:** 10.3390/genes9070332

**Published:** 2018-07-02

**Authors:** Mafalda Cavalheiro, Pedro Pais, Mónica Galocha, Miguel C. Teixeira

**Affiliations:** 1 Department of Bioengineering, Instituto Superior Técnico, Universidade de Lisboa, 1049-001 Lisbon, Portugal; mafalda.cavalheiro@tecnico.ulisboa.pt (M.C.); pedrohpais@tecnico.ulisboa.pt (P.P.); monicagalocha@tecnico.ulisboa.pt (M.G.); 2 Biological Sciences Research Group, iBB—Institute for Bioengineering and Biosciences, Instituto Superior Técnico, Universidade de Lisboa, 1049-001 Lisboa, Portugal

**Keywords:** fungal pathogens, multidrug transporters, host-pathogen interaction, virulence

## Abstract

Fungal infections caused by *Candida*, *Aspergillus*, and *Cryptococcus* species are an increasing problem worldwide, associated with very high mortality rates. The successful prevalence of these human pathogens is due to their ability to thrive in stressful host niche colonization sites, to tolerate host immune system-induced stress, and to resist antifungal drugs. This review focuses on the key role played by multidrug resistance (MDR) transporters, belonging to the ATP-binding cassette (ABC), and the major facilitator superfamilies (MFS), in mediating fungal resistance to pathogenesis-related stresses. These clearly include the extrusion of antifungal drugs, with *C. albicans CDR1* and *MDR1* genes, and corresponding homologs in other fungal pathogens, playing a key role in this phenomenon. More recently, however, clues on the transcriptional regulation and physiological roles of MDR transporters, including the transport of lipids, ions, and small metabolites, have emerged, linking these transporters to important pathogenesis features, such as resistance to host niche environments, biofilm formation, immune system evasion, and virulence. The wider view of the activity of MDR transporters provided in this review highlights their relevance beyond drug resistance and the need to develop therapeutic strategies that successfully face the challenges posed by the pleiotropic nature of these transporters.

## 1. Introduction

Species belonging to the *Aspergillus*, *Cryptococcus*, and *Candida* genera constitute the most relevant human fungal pathogens. Infections caused by these pathogens are especially severe in immunocompromised patients, particularly HIV-infected patients, cancer patients, and transplant recipients [1,2,3]. *Candida albicans* and *Candida glabrata* are the most prevalent of the pathogenic *Candida* species, being responsible for more than 400,000 life-threatening infections worldwide every year [4], as well as persistent mucosal infections [5,6]. *Aspergillus fumigatus* is the most frequent pathogenic species of the *Aspergillus* genus found to cause life-threatening pulmonary disease [7]. Central nervous system manifestations of meningitis or meningoencephalitis are recurrent manifestations associated with *Cryptococcus neoformans* infections [8].

One very resourceful feature of pathogenic fungi is the expression of multidrug resistance (MDR) transporters, which allow the development of antifungal drug resistance, being responsible for many cases of therapeutic failure [9,10]. MDR transporters belong mainly to two superfamilies, the ATP-binding cassette (ABC) and the major facilitator superfamilies (MFS). The ABC transporters have two main domains, each including a transmembrane domain (TMD) with six trans-membrane segments and a nucleotide-binding domain (NBD). This structure usually allows the transport of different molecules against an electrochemical gradient at the direct expense of ATP hydrolysis [11,12,13]. On the other hand, the transport performed by MFS transporters is driven by a proton-motive force. The MFS-MDR transporters are clustered into two families in fungi, the drug:H^+^ antiporter 1 (DHA1), including 12 transmembrane segment proteins, and the drug:H^+^ antiporter 2 (DHA2), including 14 transmembrane segment proteins [14]. Numerous molecules are proposed to be transported by MDR transporters of both superfamilies, including steroids, lipids, anti-cancer molecules, antifungals, herbicides, antibiotics, fluorescent dyes, carbohydrates, metabolites, neurotransmitters, nucleosides, amino acids, peptides, organic and inorganic anions, cations, and various Kreb’s cycle intermediates [15,16], strongly suggesting either a promiscuous nature for MDR transporters or that they may indirectly affect the accumulation of these very diverse compounds, as a consequence of the transport of their physiological substrates.

The best-studied families of fungal drug efflux pumps are those from the model yeast *Saccharomyces cerevisiae* [17], in part, because it was the first eukaryote to have its genome sequenced [18]. In this model eukaryote, the ABC transporters have been classified into three main subfamilies, namely, the pleiotropic drug resistance (PDR), MDR, and multidrug resistance-associated protein (MRP), composing a total of 21 ABC drug resistance-related transporters [17,19]. Additionally, a total of 22 MFS drug transporters were reported in the genome of *S. cerevisiae* [20].

Genome sequencing has revealed that there is a wide repertoire of predicted ABC drug efflux pumps among pathogenic fungal species. However, only a few of these proteins have been functionally characterised, including those reported to be involved in multidrug resistance in human pathogens, such as *Candida albicans* [21,22,23], *Candida glabrata* [24,25,26,27], *A. fumigatus* [28,29], and *C. neoformans* [30,31]. Gaur et al. [32] constructed a complete inventory of ABC proteins in the genome of *C. albicans*, based on sequence similarities with ABC systems in other living organisms, and they found that *C. albicans* possesses 28 putative ABC proteins. The genome of the second most prevalent *Candida* species, *C. glabrata*, is predicted to have approximately two-thirds the number of ABC transporters predicted for *C. albicans* (18) [33]. Otherwise, much larger numbers of ABC proteins are predicted in the genomes of *A. fumigatus* and *C. neoformans.* The *A. fumigatus* genome is predicted to encode 49 ABC transporters, 35 of which are predicted to be multidrug efflux pumps [7,34]. *C. neoformans* is predicted to encode 54 ABC transporters [35].

The MFS transporters constitute the largest group of secondary active transporters, functioning as uniporters, symporters, or antiporters [16]. A subset of these transporters is involved in drug efflux [36]. Costa et al. [20] listed the DHA-MFS transporters found to occur, based on phylogenetic analysis with the model yeast *S. cerevisiae*, in the pathogenic yeasts of the *Candida* genus and also in *C. neoformans*, and *A. fumigatus*. Within the DHA1-MFS family, it was found 18, 10, 9, and 54 predicted transporters in the genome of *C. albicans*, *C. glabrata*, *C. neoformans*, and *A. fumigatus*, respectively. Regarding the DHA2-MFS family, 8, 5, 7, and 32 were found as predicted transporters in the genome of *C. albicans*, *C. glabrata*, *C. neoformans*, and *A. fumigatus*, respectively.

Although the main studied function of MDR transporters in fungi is related to the efflux of antifungal drugs, whose importance in azole resistance is reviewed herein, these transporters have different physiological roles that are also determinant for the survival of fungal pathogens in the human host. In this review, a collection of the identified physiological functions of the MDR transporters is compiled, highlighting how certain roles are necessary for the adaptation of fungi to the host niches, as well as in the fight against stresses imposed by the immune response of the host. Clues on the function of MDR transporters are also extracted from the gathered information on their transcription regulators.

## 2. MDR Transporters in Fungal Pathogens: Mediators of Azole Drug Resistance

Efflux-mediated drug resistance appears to be the most widespread mechanism of azole drug resistance among pathogenic fungal species [37,38,39]. The ABC drug efflux pumps have, in general, been linked to greater clinical significance [40]. However, and unlike what has been observed for ABC drug efflux pumps, which are widespread from bacteria to man, the MFS-MDR family appears to be strictly conserved within bacteria and fungi, turning these proteins into interesting candidates for targets for the development of new antifungal drugs [20].

The ABC transporters implicated in azole drug resistance in *C. albicans* are described as *Candida* Drug Resistance *(CDR)* genes , with *CaCDR1* encoding the best characterised multidrug transporter [41]. Together with *CaCDR1*, *CaCDR2* also contributes to azole resistance mediated by increased drug efflux [23,42]. In the non-*albicans Candida* species, the *CDR* genes have also been shown to play a role in azole resistance. In *Candida dubliniensis*, *CdCDR1* is overexpressed in the fluconazole-resistant strains [43]. In addition, *CDR1* is also induced in azole resistant *Candida parapsilosis* and *Candida tropicalis* clinical isolates [44,45,46]. In turn, the expression of the ABC transporters CgCdr1 and CgCdr2 in *C. glabrata* is related with the frequent azole resistance observed in this species [47]. Additionally, the ABC transporters CgSnq2 and CgYor1 have also been implicated in azole stress response in *C. glabrata* [26,48]. *Candida krusei*, which displays intrinsic azole resistance, harbors two ABC drug efflux pumps (CkAbc1 and CkAbc2) that are upregulated during azole stress [49]. *Candida krusei* CkAbc1 is involved in intrinsic fluconazole resistance [50] and confers fluconazole resistance through drug efflux upon overexpression in *S. cerevisiae* [51]. *CkABC2* expression was correlated with itraconazole resistance [52].

In *A. fumigatus*, the mechanisms of antifungal resistance have deserved more intensive investigations in the past ten years. Alteration of the azole target *cyp51A* is a major mechanism in clinical and environmental isolates. However, there are now also several non-*cyp51A*-mediated azole-resistant isolates in which the underlying mechanisms remain partially unsolved [53]. The reduced intracellular accumulation of drugs has also been correlated with the overexpression of MDR efflux transporter genes in this pathogen [38]. In contrast to the extensive number of genes predicted to encode transporters in *A. fumigatus* [54], there are very few studies characterizing these transporters and their relationship with multidrug resistance. However, decreased intracellular accumulation of itraconazole was verified in *A. fumigatus*, hinting for a possible participation of efflux pumps in azole resistance in this fungus. Slaven et al. [29] and Nascimento et al. [55] have characterised AfuAtrF and AfuMdr4, respectively, as ABC transporters, and correlated them with itraconazole resistance. Additionally, genes encoding the set of transporters AfuAbcA-E, AtrF, and AtrI are induced during voriconazole stress [53,56].

In *C. neoformans*, the reduced intracellular accumulation of drugs has been correlated with the overexpression of MDR efflux transporter genes [39]. The genome of this pathogen is predicted to encode nearly 86 DHA-MFS transporters and 54 ABC transporters. Nevertheless, only three efflux pumps have been related with drug extrusion, CnAfr1, CnAfr2, and CnMdr1 [30,57,58]. A recent study by Chang et al. [57] demonstrated that CnAfr1 is a crucial for azole efflux and is important for handling other xenobiotics, including cycloheximide, nocodazole, and trichostatin A. The overexpression of the *C. neoformans* ABC transporter CnAfr1 is known to underlie clinical fluconazole resistance [31], while CnMdr1 confers itraconazole resistance upon overexpression in *S. cerevisiae* [51]. However, these three efflux pumps appeared to play no clear role in susceptibility towards amphotericin B and 5-fluorocytosine.

Although transporter-mediated azole resistance has been initially associated with ABC drug efflux pumps, further insights have shown that MFS-MDR transporters also play a relevant role in this phenomenon. In fact, several transporters from the MFS-MDR family are also relevant players in clinical azole resistance phenotypes, as is the case with the *C. albicans* CaMdr1. Considered a major mediator of azole resistance, it is overexpressed in some resistant clinical isolates, underlying fluconazole resistance [59]. A similar case was also identified in *Candida dubliniensis*, with increased *CdMDR1* transcript levels associated with clinical resistance phenotypes to fluconazole, but not to ketoconazole [43,60]. Moreover, *C. parapsilosis* azole resistant clinical isolates overexpress CpMdr1 [44,45], and such a response is also observed during the in vitro induction of azole resistance [61]. Resistant clinical isolates of *C. tropicalis* were found to overexpress the CtMdr1 transporter [46,62]. The *C. albicans* MFS transporter CaFlu1 confers fluconazole resistance in *S. cerevisiae*, but it plays a secondary role in *C. albicans* [63,64].

A relevant role of MFS efflux pumps in azole response is also observed in *C. glabrata*, translated by the increased expression of CgAqr1, CgQdr2, CgTpo1_1, and CgTpo3, found to mediate clotrimazole resistance in clinical isolates [10]. These transporters were previously found to mediate azole resistance in laboratory strains as well [24,25,27,65]. Interestingly, the level of correlation between the increased expression of these transporters and the azole drug resistance was similar to that observed for the *CgCDR2* gene, thus highlighting the importance of MFS-MDR transporters in the clinical setting [10]. More recently, the MFS-MDR transporters CgTpo1_2, CgFlr1, and CgFlr2, were also found to confer azole resistance, by mediating the decreased intracellular accumulation of these drugs [65,66]. In *A. fumigatus*, the MFS transporters can potentially have a relevant role in azole resistance, since AfuMdr3 displays an increased expression in itraconazole-resistant mutants [55]. In addition, three transporters (AfuMfsA–C) are highly expressed during voriconazole stress [56].

Within the MFS-MDR transporters, only eight (CaMdr1, CaFlu1, CaNag3, CaNag4, CaNag6, CaQdr1, CaQdr2, and CaQdr3), seven (CgAqr1, CgFlr1, CgFlr2, CgTpo1_1, CgTpo1_2, CgTpo3, CgQdr2), three (CNA07070, CNC03290, and CND00440/aflT), and six (gliA, mfs56, Mdr3, and MfsA–C), were already characterised in *C. albicans*, *C. glabrata*, *C. neoformans*, and *A. fumigatus*, respectively [20].

Not only is there a wide variety of MDR transporters involved in azole resistance in several fungal pathogens, but the regulation of their expression is also diverse. The most prominent mechanism of azole resistance acquisition involves increased drug efflux pumps gene expression, which is mediated by master regulators of azole resistance. They include Tac1 and Mrr1 in *C. albicans* and related species; and CgPdr1 in *C. glabrata*. These transcription factors are subjected to gain-of-function (GOF) mutations in their protein sequence, which result in hyperactive forms, responsible for constitutive increased transcription of their target genes [44,61,67,68,69,70,71,72]. Additionally, each transcription factor regulates its own expression, increasing their own transcript levels in response to azole stress [67,73,74,75]. ABC transporters in particular have been first described as targets of CaTac1 and CgPdr1 [26,48,76,77,78], but several MFS transporters are also activated by CaMrr1 and CgPdr1 [65,66,71,72], thus reinforcing the role of multiple transporters in azole resistance pathways.

Other than mutations in transcriptional regulators, another regulatory mechanism mediating azole resistance is chromosomal abnormalities. These can include loss of heterozygosity (LOH), chromosomal aneuploidies, or increased gene copy number. Development of azole resistance by LOH events is known to occur in *C. albicans*, namely in the genomic regions containing *CaTAC1* or *CaMRR1*. Mutations in *MRR1* followed by the loss of heterozygosity, contribute to the overexpression of this gene [72]. This phenomenon also occurs to give rise to *CaTAC1* homozygous mutations because of the loss of heterozygosity in chromosome 5 [70]. Chromosomal rearrangements also contribute to the amplification of the *CaTAC1* gene [79]. In *C. glabrata*, the existence of differential chromosome configurations and segmental aneuploidies was observed in azole-resistant strains [80]. Alterations in chromosome copy number is related with *C. neoformans* resistance to azoles. In particular, CnAfr1 overexpression due to chromosome 1 duplication results in resistant populations in response to selection [81].

Although the transcriptional regulation of MDR transporters in *A. fumigatus* and *C. neoformans* have not yet been deeply studied, Hagiwara and colleagues [82] reported a novel Zn_2_–Cys_6_ transcription factor, AfuAtrR, as playing a key role in an azole resistance mechanism of *A. fumigatus*, by regulating the drug target AfuCyp51A (14-α sterol demethylase) and the putative drug efflux pump AfuCdr1B (AfuAbcC) expression. Additionally, through the screening of a library of transcription factor mutants, different transcription factors regulating the expression of efflux pumps genes in response to fluconazole exposure were identified in *C. neoformans*. These include *CnCRZ1* and *CnYAP1*, which had been previously characterised as transcription factors functioning in response to environmental stress, including thermal, hypoxic, and fluconazole, as well as oxidative stress in *C. neoformans* [83,84]. Chang and co-workers [57] determined that CnAfr1 expression was positively regulated by CnCrz1 and CnYap1 in response to fluconazole.

## 3. MDR Transporters in Fungal Pathogens: Mediating the Transport of Physiological Substrates as a Way to Adapt to Host Niches

Fungal pathogens have different strategies to colonize, survive, and infect the human host. Each species has preferential niches, developing specific mechanisms that allow adaptation to each microenvironment and evasion from the immune system. For instance, *C. albicans* exhibits different morphologies to better adapt to each niche, for example switching from the hyphal morphology used to penetrate host epithelial cells to the yeast shape to circulate in the bloodstream [85]. The preferential niches for *C. albicans* and other *Candida* species include the oral cavity, the skin, the urogenital, and gastrointestinal tracts, whose infection might lead to disseminated disease [85,86]. In the case of the *Cryptococcus* species, the preferable niches of the human host include the pulmonary tissue and the central nervous system. The establishment of a *Cryptococcus* invasive disease often starts with infection of the lung tissue, followed by invasion of the bloodstream, and subsequent dissemination of *Cryptococcus* cells to the central nervous system [87,88]. Likewise, *A. fumigatus* infects primarily the lungs, disseminating from there to any possible organ [89].

In each specific niche, different stresses like heat shock, osmotic, oxidative and nitrosative stresses, pH variations, and hypoxia might be encountered, forcing the development of stress resistance mechanisms by fungal pathogens [90]. Given the different stresses and different nutrient availability, fungal pathogens must have increased flexibility to adapt and respond to the environment. This wide capacity of metabolic adaptation and resistance to stress also promotes virulence in fungal pathogens, as it lessens their vulnerability to the surrounding environment [86].

In the oral cavity, different factors protect the host from the presence of fungal pathogens. First of all, different cell types are ready to identify them, including polymorphonuclear leukocytes, monocytes/macrophages, non-major histocompatibility complex (MHC)-restricted CD8^+^ T cells, and oral epithelial cells [91]. Upon the presence of *Candida* cells, the epithelial cells from the oral mucosa induce expression of a nitric oxide synthase, which leads to increased levels of nitric oxide believed to have a candidacidal activity, helping in the resistance to mucosal candidiasis [91]. These cells are also able to secrete different antimicrobial peptides, including members of the β-defensin family [92]. Additionally, the saliva has powerful antimicrobial peptides, such as histatins. These are known to promote cell cycle disturbance, increased intracellular reactive oxygen species (ROS), and loss of cell volume in *C. albicans*, ultimately leading to the apoptosis of yeast cells. Histatin-1, -3, and -5 are produced by acinar cells present in human salivary glands [93].

The lungs constitute the first site for *Cryptococcus* infection. This niche also has its strategies of protection against invasion. As a first defense mechanism of the innate immune system is the presence of bronchial and alveolar Type I and Type II epithelial cells that, besides forming a physical barrier, also release several cytokines and chemokines to activate an immunologic response [94]. Also important for this response is the presence in the lung tissue of inflammatory monocytes, alveolar macrophages, dendritic cells, and neutrophils [95,96]. M1 macrophages produce ROS and reactive nitrogen species (RNS), including nitric oxide, known to have anti-cryptococcal properties [96]. *Aspergillus fumigatus* also enters the host by inhalation, facing the action of alveolar macrophages, neutrophils, and dendritic cells [97,98].

In turn, the vaginal mucosa is a low pH environment with the presence of weak acids, like acetic and lactic acids [99,100], protected by *Candida*-specific cell-mediated immunity. This immunity is mediated by T helper (Th) 1-type responses [101]. Additionally, the established microbiome present in this niche is also responsible for its protection. A good example is the role of *Lactobacillus crispatus* in this context, found to mediate the immune response of vaginal epithelial cells deployed in the presence of *C. albicans* [102].

Although generally associated to multidrug resistance, some MDR transporters have been found to contribute to the adaptation of fungal pathogens to human host niches. This role of MDR transporters seems to be linked to a general function of cellular detoxification, given their ability to catalyse the efflux of antifungal drugs, but also other toxic metabolites present mainly in the stationary phase of cellular growth [103]. Interestingly, the ability to control the concentration of some of these physiological substrates is likely to confer a selective advantage when growing, both as commensals and pathogens in human host niches.

For example, the *C. albicans* ABC transporter Cdr1 was found to transport steroids. Accumulation assays with radiolabelled β-estradiol and corticosterone have demonstrated the ability of CaCdr1, expressed in the AD1234568 (AD)-*CDR1 S. cerevisiae* strain, to enhance the efflux of these human steroids, that appear to compete with drugs such as cycloheximide, O-phenanthroline, chloramphenicol, fluconazole, or rhodamine 123 as Cdr1 substrates [104]. However, the transport of [^3^H]-β-estradiol was not affected by the truncation of domain 12 of CaCdr1, a mutation that affects drug transport, suggesting that Cdr1 displays different binding sites for these different substrates [105]. Interestingly, increased incidence of vulvovaginal candidiasis has been linked to the presence of elevated estrogen levels during pregnancy or to the presence of exogenous estrogens from oral contraceptives [106,107,108,109]. The effect of β-estradiol or ethynyl estradiol, but not α-estradiol or estriol, on the formation of germ tubes has been reported, revealing the specificity of such an induction [110,111]. *Candida albicans* strain SC5314 and the vaginal isolate GC15 in Roswell Park Memorial Institute (RPMI)-free supplemented with supraphysiological (10^−5^ M) or physiological (10^−10^ M) concentrations of 17-β-estradiol and estradiol, respectively, during different time points, exhibit the upregulation of *CaCDR1* and *CaCDR2*. When both genes were disrupted, the *C. albicans* cells exposed to 17-β-estradiol had a decreased germ tube formation [111]. These evidences point out the importance that MDR efflux pump-mediated resistance to chemical compounds present in human body niches have in the success of fungal infections.

In *S. cerevisiae*, sterol uptake requires the involvement of the ABC transporters Pdr11 and Aus1. These are necessary for the nonvesicular movement, from the plasma membrane to the endoplasmatic reticulum, a movement needed for the sterol ester synthesis required for sterol uptake [112]. The *S. cerevisiae* Upc2 transcription factor is known to induce the *AUS1* and *PDR11* expression under anaerobiosis conditions, when the sterol uptake is essential. The absence of these two genes leads to defects in sterol uptake [113]. Although, in *S. cerevisiae*, no connection to MDR has been observed for these transporters; in *Cryptococcus gattii*, Pdr11 was found to be necessary for fluconazole resistance in the VGII clinical strain [114]; and *C. glabrata* Aus1 has been linked to sterol transport associated with the presence of azole drugs, being upregulated in anaerobic conditions [115]. In fact, sterol uptake was found to be used by *C. glabrata* strains defective in ergosterol [116] and in *C. glabrata* clinical isolates [117]. In fact, upon the use of azoles, which affect the production of ergosterol, the sterol uptake response is activated as a resistance mechanism that allows *C. glabrata* cells to survive ergosterol defects with host sterols, like cholesterol [118]. Sterol uptake might also be necessary for *C. glabrata* virulence, as Aus1 is necessary in a mice model of disseminated infection, being significantly upregulated in such conditions [115]. Therefore, sterol uptake is an important strategy developed by *C. glabrata* to survive in the human host, ensuring its virulence and antifungal resistance in mucosal niches.

Major facilitator superfamilies multidrug transporters have also been found to contribute to lipid homeostasis in *C. albicans* and *C. glabrata*. The MFS-MDR transporters Qdr1, Qdr2, and Qdr3 of *C. albicans* were found to be localized in the plasma membrane, with preferential localization of Qdr1 and Qdr2 in lipid rafts. These transporters do not participate in the resistance to any type of known antifungals [119], although their homologs in *C. glabrata* and in *S. cerevisiae* are recognized drug transporters [25,120,121,122]. Nevertheless, they seem to have other important functions for the survival of *C. albicans.* When absent, *CaQDR1*, *CaQDR2*, or *CaQDR3* lead to the accumulation of phosphatidylinositol and phosphatidylserine, as well as of sphingolipids, thus indicating a role of these transporters in lipid homeostasis [119]. On the other hand, the *C. glabrata* Tpo1_2 MFS-MDR transporter has also been linked to fatty acid and sterol homeostasis, especially during biofilm formation. The absence of *CgTPO1_2* upon biofilm formation leads to a decrease of very long fatty acids. Moreover, ergosterol content in the *∆cgtpo1_2* deletion mutant was found to be 30% higher than in its parental strain [123]. This physiological function of lipid transportation in *C. glabrata* seems to be important for to the adaptation to low pH environments with organic acids, as alterations in the membrane lipid composition have been described as a mechanism to surpass the stress on such environment [124].

The CgTpo1_2 homolog, CgTpo1_1, is also an MFS drug transporter with an interesting proposed role in the survival of *C. glabrata* in the oral cavity. One of the mechanisms used by the host to resist candidiasis is the release of the antimicrobial peptide histatin-5 in the saliva [93], as described above. Interestingly, the CgTpo1_1 transporter was found to be necessary for the resistance of *C. glabrata* to this antimicrobial peptide [123]. Another similar player in histatin-5 resistance is the *C. albicans* Flu1 MFS transporter involved in the resistance towards fluconazole, ketoconazole, and itraconazole [63]. In fact, the absence of *CaFLU1* significantly defects cellular growth and biofilm formation upon the exposure to this antimicrobial peptide [125]. Both studies give evidence of the role of MDR transporters’ action against antimicrobial peptides, a usually effective weapon of the human immune system against microbial infections.

The roles of the Ste6 ABC transporter of *S. cerevisiae* are also different and interesting. This transporter was found to be responsible for the secretion of a lipopeptide mating pheromone, designated a-factor, but also for the resistance of this species to valinomycin, an antibiotic peptide [126]. Interestingly, *C. albicans* homolog of Ste6, designated Hst6, was found to be able to complement the role of *S. cerevisiae* Ste6 in a *∆scste6* deletion mutant [127]. Nevertheless, its specific function in *C. albicans* remains to be discovered, as this species is not known to secrete mating pheromones. On the other hand, the Ste6 homolog in *C. neoformans* has been shown to be involved in the mating process of this pathogen [128]. In turn, *A. fumigatus* Afu3g03430, which is also similar to *S. cerevisiae* Ste6, has been indicated as being involved in iron uptake [129,130,131]. Iron uptake is an important function for the survival of any fungal pathogen, as this cofactor is necessary for several enzymatic reactions and is withheld in the human host as a protective mechanism against microbial infections [132].

Another example of the importance of MDR transporters in the adaptation to human niches is their role in polyamine resistance. Polyamines like spermine, spermidine, and putrescine are essential organic polycations, which are usually involved in the regulation of nucleic acid and protein synthesis, as well as cell growth [14]. The plasma membrane MFS-MDR transporters, Tpo1–4 and Qdr3, were the first identified as important players in the resistance to polyamines in *S. cerevisiae* [133,134,135]. In *C. glabrata*, MFS-MDR transporters have also been found to contribute to the resistance to polyamines, which may become toxic above certain concentrations, such as those found in the human urogenital tract [136]. The prevalence of *Candida* species in this niche is likely facilitated by two homologs of ScTpo1 in *C. glabrata*, CgTpo1_1 and CgTpo1_2, which have been identified as determinants in spermine resistance [65]. In turn, the CgTpo3 transporter, known to be involved in *C. glabrata* azole resistance, presents the same physiological role as its *S. cerevisiae* homolog, Tpo3, in polyamine resistance [24].

Since the vaginal tract is characterised by an acidic pH of about 4, as well as the presence of weak acids, like acetic and lactic acids [99,100], the ability to tolerate the stress induced by weak acids is crucial for *Candida* colonization and successful infection. Interestingly, *S. cerevisiae* MFS-MDR transporters, like Aqr1 and Qdr1, are important determinants in short-chain monocarboxylic acid resistance [120,137]. *S. cerevisiae* Aqr1 has also been implicated in the excretion of homoserine, threonine, alanine, glutamate, and aspartate, probably through vesicles, releasing the amino acids to the extracellular environment through exocytosis [138]. The homolog of *S. cerevisiae* Aqr1 in *C. glabrata*, CgAqr1, has been linked to flucytosine and clotrimazole resistance, as well as resistance against acetic acid [27]. Such a role in the resistance to acetic acid is also performed by the MFS-MDR CgDtr1 and CgTpo3 transporters [139,140], being the later controlled by the CgHaa1 transcription factor, known to regulate the response of *C. glabrata* to acetic acid [140]. These different roles of MFS transporters [14,20] point out their importance for the survival of such pathogens in acidic human microenvironments.

## 4. MDR Transporters in Fungal Pathogens: Playing a Role in Virulence, Biofilm Formation, and Phagocytosis Evasion

**Figure 1:**
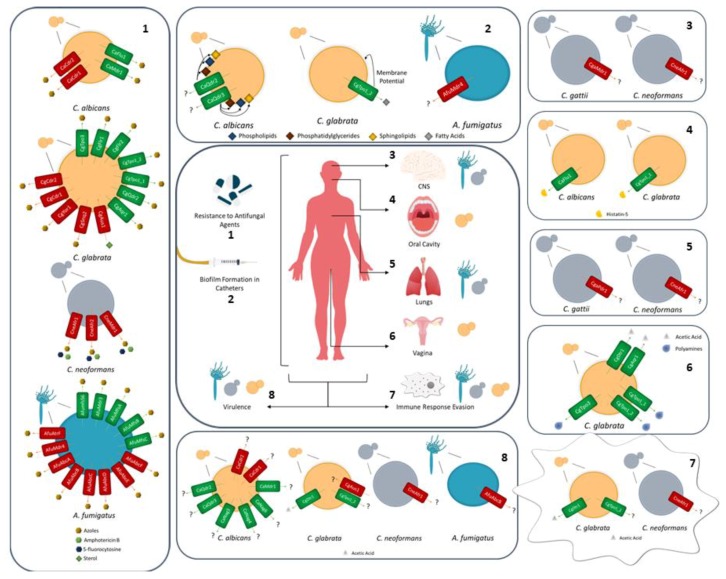
The role of multidrug resistance (MDR) transporters in the survival of *Candida*, *Cryptococcus*, and *Aspergillus* species upon infection in the human host. (1) MDR transporters involved in multiple azole resistance; (2) MDR transporters necessary for biofilm formation; (3) MDR transporters involved in *Cryptococcus* species virulence in the central nervous system (CNS); (4) MDR transporters involved in the efflux of histatin-5 in the oral cavity; (5) MDR transporters involved in *Cryptococcus* species virulence in the lungs; (6) *Candida glabrata* MDR transporters involved in polyamine or acetic acid export for the survival in the vaginal tract; (7) MDR transporters involved in the fight against the immune response of the host; and (8) MDR transporters known to be essential for normal virulence of fungal pathogens. The central picture summarizes the niches of infection in which the referred fungal pathogens are found. ATP-binding cassette (ABC) transporters are highlighted in red and major facilitator superfamilies (MFS) transporters are highlighted in green.

To persist in the human host, fungal pathogens need to survive in the first site of infection, and disseminate to other niches of the host. To achieve this goal, several barriers imposed by the niches need to be surpassed, as well as the ones imposed by the immune response (Figure 1). To face and resist such offenses, fungal pathogens develop strategies of survival, including biofilm formation, macrophage evasion mechanisms, and virulence factors, where the role of given MDR transporters becomes important.

For example, similarly to the *S. cerevisiae* Pdr5 and Yor1 ABC transporters [17], *C. albicans* CaCdr1, CaCdr2, and CaCdr3 have been shown to play a role in phospholipid translocation when expressed in *S. cerevisiae*. CaCdr1 and CaCdr2 conduct an in-to-out movement of phospholipids through the plasma membrane bilayer in an energy-dependent manner, while CaCdr3, which is not involved in antifungal drug resistance, produces the opposite movement, from the outer to the inner leaflet of the plasma membrane [141]. The transport of phospholipids by Cdr1 may be extremely important for *C. albicans* evasion to the host immune system, as some phospholipids have been identified as lipid antigens and precursors of lysophospholipids, also known to be lipid antigens recognized by human natural killer T lymphocytes [142,143]. For instance, to avoid identification by the host immune response, *C. albicans* relies on CaQdr2 and CaQdr3 transporters, given that they are necessary for the maintenance of phosphatidylinositol, which is a lipid antigen recognized by the CD1 antigen-presenting cells from the host [144]. Interestingly, the absence of *CaQDR2* and *CaQDR3* genes have shown to attenuated *C. albicans* virulence in a murine model of hematogenously disseminated candidiasis [119].

When fungal evasion is not effective, pathogens are phagocyted and a new environment is set upon them, to which they have to adapt to survive. Different host cells are able to perform phagocytosis, including monocytes/macrophages, neutrophils, and dendritic cells [145,146]. All these lead to the formation of phagolysosomes that attempt to kill pathogens by producing ROS and RNS, activating the action of proteases and decreasing the pH, resorting to K^+^ fluxes [146]. *Candida* species display an activation of oxidative stress response in this environment, as well as other survival mechanisms [147]. In particular, *C. glabrata* cells are known to be able to resist and proliferate inside mammalian macrophages for a very long time [148]. Together with other more specific mechanisms, *C. glabrata* is known to count with two transporters from the MFS-MDR family, CgAqr1 and CgDtr1, which are responsible for the resistance to weak acids and oxidative stress [27,139]. CgAqr1 was found to confer resistance to acetic acid [27], like its homolog in *S. cerevisiae* [137], and CgDtr1 was identified as an acetic acid exporter, allowing the resistance to this acid and oxidative stress [139]. It is likely that the role of CgDtr1 in weak acid and oxidative stress resistance underlies its contribution to the survival of *C. glabrata* in the macrophages and, indirectly, to increase virulence. Indeed, the *CgDTR1* expression is upregulated in cells phagocytozed by *Galleria mellonella,* hemocytes and the overexpression of this gene leads to an increase in the survival of *C. glabrata* cells in *G. mellonella* hemocytes. Furthermore, CgDtr1 was found to be necessary for the full virulence of *C. glabrata* in the infection model *G. mellonella* [139].

*Cryptococcus neoformans* survival in phagocytic cells also depends on its reaction to the new environment with low pH, ROS, and nitric oxide [96]. Upon *C. neoformans* phagocytosis by macrophages in vitro, the overexpression of the *CnAFR1* gene takes place [39]. The Afr1 ABC transporter is known for the in vitro and in vivo resistance to fluconazole [31,39,58], but has also been linked to the virulence of *C. neoformans*, in intravenous and in inhalation mouse models [39]. Although not helping to avoid phagocytosis, CnAfr1 delays the maturation of the phagolysosome, which exhibits less acid vacuoles. CnAfr1 seems to interfere with the pathway of phagolysosome maturation, as the early endosome marker Rab5 and late marker Rab7 are less detected upon the overexpression of the *CnAFR1* gene [149]. Moreover, Goulart and colleagues have reported the upregulation of other ABC transporters in *C. neoformans* cells phagocyted by peritoneal macrophages [150], highlighting the importance these transporters within the study of the *Cryptococcus* species survival in the host.

*Cryptococcus gattii* seems to count with drug transporters for its full virulence in the brain and the pulmonary tissue, like the MFS Mdr1 and the ABC Pdr11 transporters, determinants of *C. gattii* MDR [58,114]. The *MDR1* gene was found to be upregulated in cells recovered from the brain and lungs of infected mice, while *PDR11* was overexpressed in cells recovered from the lungs of infected mice [151]. Both Mdr1 and Pdr11 were proposed to play an additional role *C. gattii* virulence and adaptation to the host environment, although the exact mechanism underlying this observation is unclear.

Likewise, *A. fumigatus* infects primarily the lungs disseminating to any possible organ [89]. Such infections are believed to make use of the 49 ABC-like genes and 278 MFS-like genes present in this species [54], which, given their elevated number, indicate a major role in *A. fumigatus*. From all these, some have been identified as antifungal resistant players [28,29,55,56], but having additional roles in expelling toxic molecules [54]. abcA and abcB are two *A. fumigatus* transporters, with a high similarity to the *S. cerevisiae* Pdr5 drug efflux pump. abcB is necessary for full virulence of *A. fumigatus* in the *G. mellonella* model of infection and the overexpression of the *abcA* gene leads to an augmentation of virulence in the presence of voriconazole, in the same infection model [152].

The *C. albicans* Mdr1 MFS transporter is well known for its role in MDR [9,153,154]. Nevertheless, this transporter has been identified as necessary for full virulence in *C. albicans* in immunocompetent and immunocompromised mice models [155], although its specific functions in virulence have not yet been assessed. Moreover, the CaNag3, CaNag4, and CaNag6 MFS transporters are necessary for the full virulence of *C. albicans* in a mouse model [156].

Several MDR transporters have also been shown to have roles in biofilm formation, an important feature that allows the persistence of fungal pathogens in the host [157]. One specific case is the activity displayed by CaQdr2 and CaQdr3 in the architecture of *C. albicans* biofilm, which, although not contributing for the total biomass produced, help to shape the structure of the biofilm [119]. In *C. glabrata*, the CgTpo1_2 has been highlighted as an important player in biofilm formation, being necessary for the normal expression of *CgALS1*, *CgEAP1*, and *CgEPA1* genes encoding adhesins. Its role in biofilm formation seems to be linked to its importance in fatty acids homeostasis, as it affects the incorporation of very long chain fatty acids, which are usually found in *C. glabrata* biofilms [123]. In turn, *A. fumigatus* Mdr4 transporter has been identified as upregulated upon voriconazole exposure and biofilm formation conditions [129,158], indicating a possible role in the development of biofilm. Interestingly, GOF mutations in the multidrug resistance transcription factor Pdr1 were also found to be required for increased virulence and biofilm formation, by controlling the expression of the adhesion encoding gene *EPA1* [159,160].

## 5. Hints on the Function of MDR Transporters Based on Transcription Regulation

Recently, clues on the physiological roles of MDR transporters, including the transport of lipids, ions, and small metabolites, have emerged from transcriptional regulation data, linking these transporters to important pathogenesis features such as resistance to host niche environments, biofilm formation, immune system evasion, and virulence. In this section, all available information on the transcriptional regulation of ABC/MFS-MDR transporter genes in the human pathogens *C. albicans* and *C. glabrata* is reviewed. Although a similar analysis would be very interesting for *A. fumigatus* and *C. neoformans*, the available information is very limited and scattered. On the contrary, information on *C. albicans* and *C. glabrata* has been recently gathered in the PathoYeastract database (www.pathoyeastract.org) [161], enabling the analysis of MDR transporter regulation, not only in the multidrug resistance context, but also within the perspective of their physiological roles and their implication in other pathogenesis-related phenotypes.

**Figure 2:**
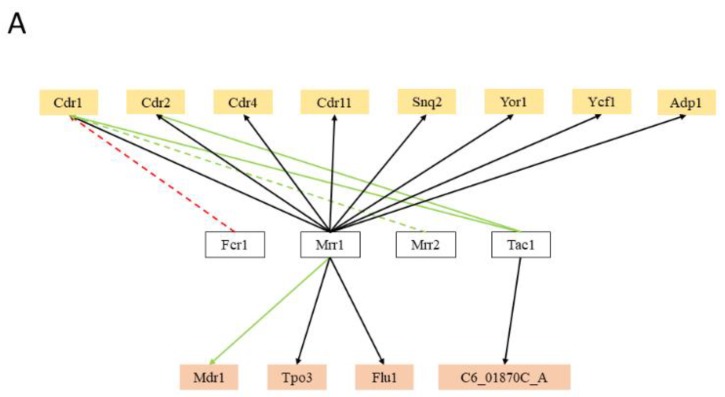

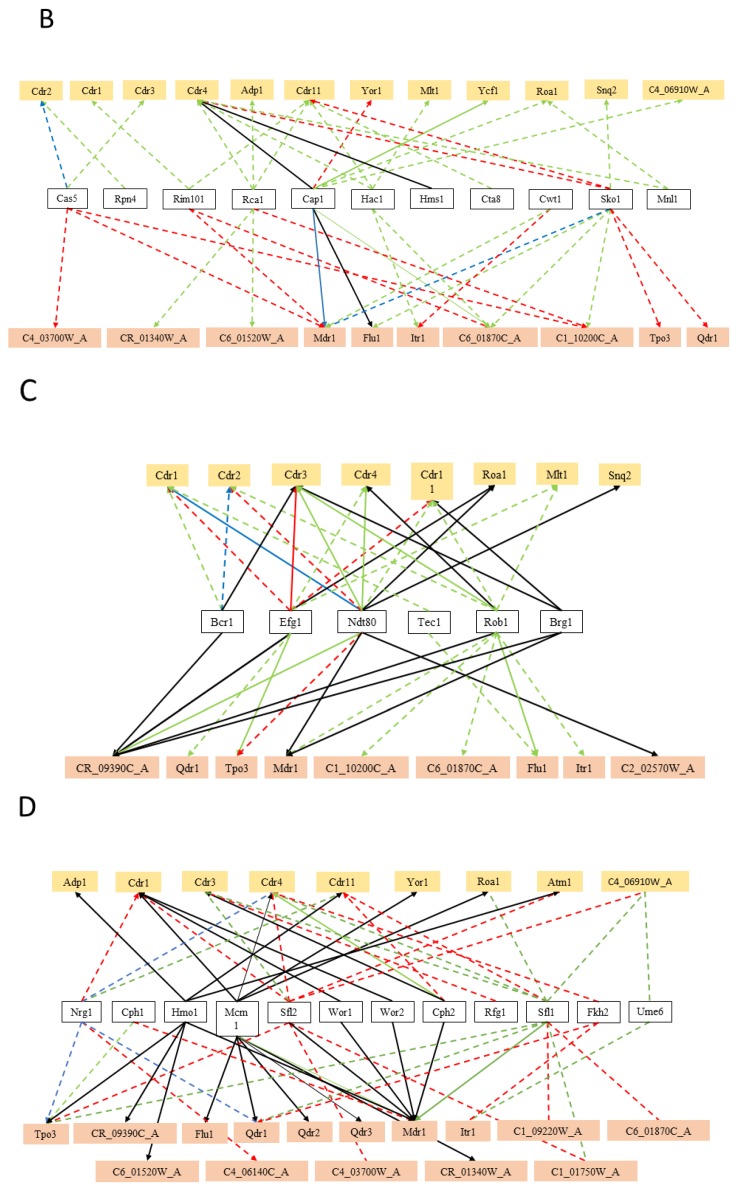

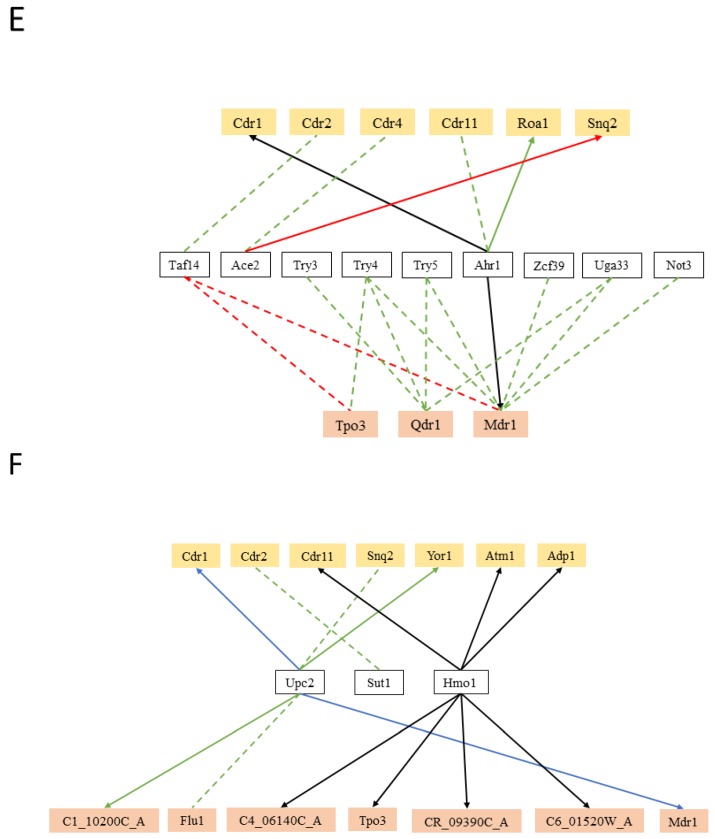
Transcriptional regulatory networks that control the expression of MDR transporter genes in *C. albicans*, considering the subgroups of transcription factors known to be involved in multidrug resistance (**A**); in stress response (**B**); biofilm formation (**C**); cell-cycle/morphology (**D**); cell-wall dynamics (**E**); and lipid (**F**). The displayed regulatory associations are according to the data present in the Pathoyeastract database (http://pathoyeastract.org/) [161]. The ABC transporters are highlighted by the yellow colour and the MFS transporters are highlighted by the orange colour. Arrows indicate the experimental basis of the documented regulatory associations, either expression evidence (dashed line) or DNA-binding evidence (filled line). Green, red, blue, or black arrows indicate a positive, negative, positive and negative, or unspecified association, respectively.

Besides the expected role of the MDR transcription factors, transcription factors controlling stress response, cell cycle, cell wall dynamics, biofilm formation, lipid and carbohydrate metabolism, and nutrient availability have been found to regulate the expression of the MDR transporter encoding genes in *C. albicans*. In fact, the number of regulatory associations with MDR transporter genes involving transcription factors virtually not related to MDR is much higher than that involving recognized MDR transcription factors. Surprisingly, with regards to multidrug resistance, only 36% (12 out of 33) of the MDR-related transporters in *C. albicans* are reported to be regulated by at least one MDR-related transcription factor, more specifically, 57% (8 out of 14) of the ABC transporters and nearly 21% (4 out of 19) of the MFS-MDR transporters (Figure 2A). Expectably, the transcription factor CaMrr1, a multidrug resistance regulator in *C. albicans*, was found to regulate 11 out of the 12 MDR transporters reported to be regulated by at least one MDR-related transcription factor, including the MFS-MDR transporter, CaMdr1, a key player in the acquisition of azole resistance in clinical isolates [71]. Transporters that have been clearly linked to resistance of a wider range of drugs are indeed those that are regulated by a higher number of MDR transcription factors. This is the case for *CaCDR1* and *CaCDR2*, which are the main ABC transporter genes responsible for azole drug resistance in *C. albicans*. *CaCDR1* is regulated by CaFcr1, CaMrr1, CaMrr2, and CaTac1, whereas *CaCDR2* is reported to be regulated by CaMrr1 and CaTac1. In particular, the *CaCDR1* regulation appears to be highly complex, being controlled by at least 19 transcription factors not only related to MDR, but also with biofilm formation, stress response, cell-wall dynamics, carbohydrate metabolism, cell-cycle, and lipid metabolism. Besides CaMrr1, *C. albicans* carries yet another major regulator of multidrug resistance transporters, CaTac1, which is described as the major factor needed for the regulation of *CaCDR1* and *CaCDR2* [76]. Nevertheless, contrasting with CaMrr1, CaTac1 appears to regulate no other MDR transporter genes besides *CaCDR1* and *CaCDR2* (Figure 2A).

It is surprising the number of stress-related transcription factors reported to regulate MDR transporters in *C. albicans*. Nearly 67% (22 out of 33) of the *C. albicans* MDR transporters have been found to be regulated by at least one stress-related transcription factor, with the transcription factors CaCap1 and CaSko1 regulating the highest number of transporters (eight and nine, respectively) (Figure 2B). These two transcription factors are known to be required for oxidative stress tolerance in *C. albicans* [162,163], which is a vital process for this pathogen to survive in healthy hosts, especially during phagocytosis. Additionally, the transcription factor CaRim101 was found to regulate the expression of a considerable number of MDR transporter genes (7). This regulator plays a crucial role in pH-response [164], and the Rim101 pathway is required for host-pathogen interactions, as it regulates the expression of genes that stimulate host cell damage [165]. Among other stress-related transcription factors that were found to control MDR transporter genes are CaCta8, an essential transcription factor that mediates heat shock response [166]; CaRpn4 and CaHac1, key regulators of unfolded protein response (UPR), which is a crucial phenomenon for cellular protein homeostasis maintenance, which is often lost when cells are under stress such as that induced by antifungals [167]; and Cas5, a zinc finger transcription factor that controls the response to cell wall stress, including those induced by echinocandins [168], but also the response to membrane stress exerted by the azole antifungal drugs [169].

Transcription factors related to biofilm formation were also found to regulate a significant number of MDR transporters in *C. albicans* (nearly 52%, 17 out of 33) (Figure 2C). Nobile et al. [170] described and analysed the transcriptional network controlling the biofilm formation in *C. albicans*, whose biofilms are a major source of medical device-associated infections. They demonstrated that CaBcr1, CaTec1, CaEfg1, CaNdt80, CaRob1, and CaBrg1 are the major players in the transcriptional network controlling the biofilm development in this human pathogen, including many MDR transporters. In fact, genes encoding for drug efflux pumps had been previously been reported in biofilms to be differentially regulated during development, as well as upon exposure to antimicrobial agents, including *CaCDR1*, *CaCDR2*, *CaMDR1*, *CaNAG3*, and *CaNAG4* [171,172,173]. CaRob1 and CaNdt80 were found to regulate the highest number of MDR transporters (nearly 65%, 11 out of 17 each), whereas CaTec1 was found to regulate a smaller portion of those (approximately 12%, 2 out of 17). These associations corroborate the observation that efflux pump-mediated multidrug resistance is an important trait of biofilm cells [174].

In *C. albicans*, the ability to undergo morphological switching from yeast cells to hyphae, in response to various environmental signals, is an important virulence factor that contributes to biofilm formation, invasion and dissemination of *Candida* in host tissues, and resistance to macrophage and neutrophil engulfment [171,175,176]. Interestingly, cell-cycle/morphology related transcription factors were found to regulate the highest portion of MDR transporters, 72% (24 out of 33), including the *CDR* genes and *MDR1* (Figure 2D). In fact, despite the fact that cell-cycle related transcriptional regulators have never been demonstrated to be crucial for efflux pump expression, a positive correlation between the level of antifungal drug resistance and the ability to form hyphae in the presence of azole drugs has been identified [176]. For instance, Mcm1, which is an essential transcription factor in *C. albicans* crucial for morphogenesis [177], was found to regulate the expression of several MDR transporters (10 out of 24), including *CaCDR1* and *CaMDR1*. CaMcm1 was previously demonstrated to be dispensable for *CaMDR1* upregulation in response to H_2_O_2_ , but was required for full *CaMDR1* induction by benomyl [177]. The transcription factors CaSfl1 and CaSfl2, two homologous heat shock factor-type transcriptional regulators that antagonistically control morphogenesis in *C. albicans*, while being required for full pathogenesis and virulence [178], were also found to regulate a number of MDR transporters (11 and 9, respectively). Finally, CaWor1 and CaWor2 are transcriptional regulators of *C. albicans* opaque cell formation, and were also found to regulate MDR transporters.

Also, cell-wall dynamics related transcription factors regulate MDR transporters in *C. albicans* (9), including the *CDR* gene family and *CaMDR1* (Figure 2E). The Try transcription factors and Taf14, Ahr1, Uga33, and Zcf39 are all reported regulators of *C. albicans* yeast form adherence, and they were found to regulate at least one MDR transporter.

Additionally, CaUpc2, the master regulator of the ergosterol biosynthesis (*ERG*) genes [179], and CaHmo1, which was also shown to bind promoters of ergosterol metabolism genes [180], regulate the expression of 13 MDR transporters (Figure 2F). The regulation of ABC drug efflux pumps by the lipid metabolism related transcription factor, is likely to be due to the activity of some of these MDR transporters in phospholipid translocation or ergosterol transport. It is not likely that all drug pumps involved in multidrug resistance are phospholipid translocators. For instance, *CaMDR1* shows no detectable phospholipid exchange activity [181]. However, the observation that these transcription factors also control the expression of MDR-MFS transporters, suggest that they may also play a role in this lipid metabolism. This hypothesis is consistent with the observation that the deletion of MFS-MDR genes *CaQDR1*, *CaQDR2*, and *CaQDR3* [119], or *CgTPO1_2* [123], does affect lipid composition.

**Figure 3:**
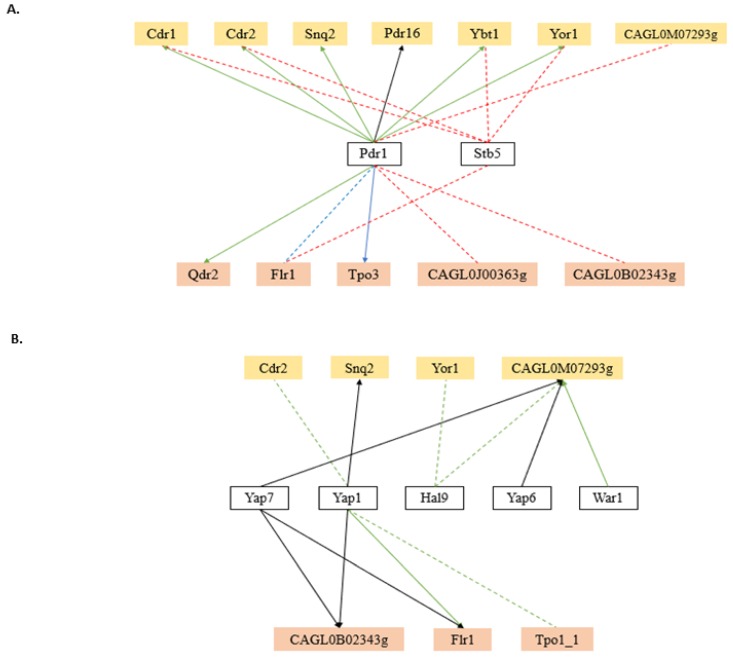
Transcriptional regulatory networks that control the expression of MDR transporter genes in *C. glabrata*, considering the subgroups of transcription factors known to be involved in multidrug resistance (**A**) and in stress response (**B**). The displayed regulatory associations are according to the data present in the Pathoyeastract database (http://pathoyeastract.org/) [161]. ABC transporters are highlighted by the yellow colour and MFS transporters are highlighted by the orange colour. Arrows indicate the experimental basis of the documented regulatory associations, either expression evidence (dashed line) or DNA-binding evidence (filled line). Green, red, blue, or black arrows indicate a positive, negative, positive and negative, or unspecified association, respectively.

In the case of *C. glabrata*, only a few regulators of MDR transporter genes have yet been unveiled, reflecting the fact that the study of transcriptional regulation in this yeast is still in its infancy, especially in biological processes beyond drug resistance. The transcription factor CgPdr1 is described as the master regulator of multidrug resistance in this organism, regulating the expression of several MDR transporter genes [48]. CgPdr1 is thought to form a heterodimer with CgStb5, as it happens in the closely related *S. cerevisiae*. The overexpression of *CgSTB5* in *C. glabrata* represses azole resistance, while its deletion produces a shy intensification in resistance. Expression analysis assays established that CgStb5p shares many transcriptional targets with CgPdr1, but, unlike the second, it is a negative regulator of pleiotropic drug resistance [68,182]. These two MDR-related transcription factors were found to regulate a total of 12 MDR transporters (seven ABC and five MFS) (Figure 3A).

Besides the expected role of MDR transcription factors, stress response transcription factors have been found to regulate the MDR transporter encoding genes in *C. glabrata*. Five stress-responsive transcription factors are reported to regulate the expression of at least one MDR transporter gene (Figure 3B). CgYap1 is the major regulator of oxidative stress response genes in *C. glabrata* [183], and it was also demonstrated to induce the expression of multidrug transporters [184]. As antifungals induce the endogenous production of ROS, thus inducing oxidative stress response mediated by CgYap1, it is likely that CgYap1 targets the MDR transporters that play a role in the extrusion of oxidative stress generating molecules from the cells. Otherwise, CgYap7 is a transcriptional repressor of nitric oxide oxidase and also regulates the iron–sulfur cluster biogenesis [185]. The remaining transcription factors present in this group were not yet characterised. However, they are thought to be involved in salt tolerance (CgHal9 and CgYap6) and weak acid response (CgWar1), based on the function of their *S. cerevisiae* homologs. It is interesting to note that the ABC transporter encoding ORF *CAGL0M07293g*, although uncharacterised, is reported to be regulated by CgYap7, CgYap6, CgHal9, and CgWar1 transcription factors. This is in accordance with its predicted function as a weak-acid-inducible multidrug transporter required for weak organic acid resistance, based on the function of its closest *S. cerevisiae* homolog ScPdr12.

## 6. Conclusions and Perspectives.

MDR transporters are undoubtedly necessary players for the successful survival of fungal pathogens in the human host (Table 1 and Table 2). As reviewed herein, their prominent role in MDR has a real impact in the clinical acquisition of drug resistance, allowing these pathogens to persist even upon treatment with different antifungal agents. Indeed, within the characterised MDR transporters, 88.5% and 71.4% of the ABC and MFS, respectively, have been shown to contribute to drug resistance, suggesting that this is their most relevant feature.

This review, however, highlights the observation that both ABC and MFS-MDR transporters contribute to the ability of fungal pathogens to colonize the host and evade host induced defenses, by executing other functions not directly connected with MDR. The study of the transcriptional networks controlling the expression of drug transporters in *C. albicans* and *C. glabrata* points towards possible links between MDR transporters and several cellular processes, including stress response, morphological switching, cell wall, and lipid homeostasis. Indeed, mounting evidence suggests that the function of MDR transporters goes well beyond their traditional role in drug resistance. Clues on what may be the physiological role of these transporters, suggest that their natural activity is not linked to the transport of chemical compounds that are not found in nature, but rather the transport of metabolites that can be found in the natural ecosystems where pathogenic fungi thrive. Among the characterised ABC and MFS transporters, 19.2% and 42.8%, respectively have been associated to the transport of such biomolecules. Significantly, MDR transporter roles associated to phospholipid and ergosterol incorporation, as well as in the excretion of metabolites that reach toxic concentrations in host niches, such as polyamines and weak acids, establish a strong link between the MDR transporters and the survival of fungal pathogens in several human microenvironments. Additionally, a few MDR transporters have been linked to the resistance to stress imposed by the host immune system, including phagocytosis and antimicrobial peptides, such as histatin-5. Not surprisingly, a high proportion of the characterised ABC (43.5%) and MFS (39.3%)-MDR transporters were found to be required for full virulence in infection models, several of which, however, for unknown reasons. It will be interesting to ascertain the precise function of these transporters in the context of virulence.

Despite their crucial role in several aspects of pathogenesis, there is still a striking lack of knowledge about the function of the majority of the MDR transporters. Indeed, among the 241 predicted ABC drug transporters in the fungal pathogens approached in this review, only 10.8%, that is 26, have been characterised. In the case of the predicted MFS-MDR transporters, only 5.8% (28 out of 479), have been functionally analysed. This observation highlights the pressing need to invest in the study of these two families of drug transporters, with special emphasis on less studied organisms, which in this context includes *A. fumigatus*, for which only 2.1% of the MFS-MDR transporters have been characterised, or *C. tropicalis*, for which only 4.5% of the ABC drug efflux pumps have been studied.

Overall, this review gathers evidence of the multitasking capacity of MDR transporters in fungal pathogens, while highlighting their key role in the successful colonization, persistence, and virulence in the human host. The truly pleiotropic activity of the ABC and MFS-MDR transporters underlines their importance in fungal pathogenesis, and highlights them as very promising drug targets for the development of new antifungals.

## Figures and Tables

**Table 1:** ATP-binding cassette (ABC) transporters of *Candida*, *Aspergillus*, and *Cryptococcus* species focusing their roles in multidrug resistance (MDR) and physiological functions, as well as their pathogenicity and virulence features.

	Species	Total #	Characterised #	Characterised ORFs	Role in MDR	Physiological Role	Pathogenicity and Virulence Features
**ABC Proteins**	*Candida albicans*	28	4	*orf19.6000/CDR1*	Multidrug transporter of ABC superfamily	Transport of phospholipids (in-to-out direction), steroids	Induced by β-estradiol, progesterone, corticosteroid, or cholesterol; Spider biofilm induced
				*orf19.5958/CDR2*	Multidrug transporter of ABC superfamily, overexpressed in azole-resistant isolates	Transports phospholipids (in-to-out direction)	Repressed in young biofilms
				*orf19.1313/CDR3*	-	Transporter of the Pdr/Cdr family of the ATP-binding cassette superfamily; transports phospholipids (out-to-in direction); expressed in opaque-phase cells	Induced by macrophage interaction; Spider biofilm induced
				*orf19.5079/CDR4*	-	-	Rat catheter and flow model biofilm induced
	*Candida glabrata*	18	5	*CAGL0M01760g/CDR1*	Multidrug transporter of ABC superfamily, involved in resistance to azoles, expression regulated by Pdr1p, increased abundance in azole resistant strains	Expression increased by loss of the mitochondrial genome	-
				*CAGL0F02717g/CDR2 (PDH1)*	Multidrug transporter, predicted plasma membrane ATP-binding cassette (ABC) transporter; regulated by Pdr1p; involved in fluconazole resistance	-	-
				*CAGL0I04862g /SNQ2*	Predicted plasma membrane ATP-binding cassette (ABC) transporter, putative transporter involved in multidrug resistance; involved in Pdr1p-mediated azole resistance	-	-
				*CAGL0G00242g/YOR1*	Putative ABC transporter involved in multidrug efflux; gene is upregulated in azole-resistant strain	-	-
				*CAGL0F01419g/AUS1*	-	ATP-binding cassette transporter involved in sterol uptake	Necessary for *C. glabrata* virulence in a mice model of disseminated infection
	*Candida tropicalis*	22	1	*CDR1*	Induced in clinical azole resistant isolates	-	-
	*Candida parapsilosis*	19	1	*CPAR2_405290/CDR1*	Induced in clinical azole resistant isolates	-	-
	*Candida dubliniensis*	19	1	*Cd36_85210/CDR1*	Predicted multidrug transporter of ABC superfamily, involved in multidrug resistance, overexpressed in fluconazole-resistant derivatives obtained in vitro	-	-
	*Candida krusei*	9	2	*ABC1*	Upregulated during azole stress, involved in innate fluconazole resistance, confers fluconazole resistance through drug efflux upon hyperexpression in *S. cerevisiae*	-	-
				*ABC2*	Upregulated during azole stress, expression correlated with itraconazole resistance	-	-
	*Aspergillus fumigatus*	49	7	*Afu6g04360/ATRF*	Putative ABC transporter; drug efflux pump; involved in itraconazole resistance	-	-
				*Afu1g12690/MDR4*	ABC multidrug transporter; induced by voriconazole exposure in vitro and in mice; involved in itraconazole resistance	-	Biofilm growth regulated protein
				*Afu1g17440/ABCA*	ABC drug exporter; induced during voriconazole stress	-	Overexpression leads to an augmentation of virulence in the presence of voriconazole in the *G. mellonella* model of infection
				*Afu1g10390/ABCB*	Putative ABC multidrug transporter; transcript induced by voriconazole	-	Necessary for full virulence of *A. fumigatus* in the *G. mellonella* model of infection
				*Afu1g14330/ABCC*	Putative ABC transporter; induced during voriconazole stress; mutation causes increased itraconazole, voriconazole and posaconazole sensitivity	-	-
				*Afu6g03470/ABCD*	Putative *MDR1* family ABC transporter; induced during voriconazole stress	-	Biofilm growth regulated protein
				*Afu7g00480/ABCE*	Putative ABC transporter; induced during voriconazole stress	-	Biofilm growth regulated protein
				*Afu3g07300/ATRI*	Putative ABC transporter; induced during voriconazole stressmutation causes increased itraconazole and voriconazole sensitivity	-	-
	*Cryptococcus neoformans*	54	3	*AFR1*	Pump required for azole efflux and other xenobiotics, including cycloheximide, nocodazole, and trichostatin A; involved in clinical fluconazole resistance; role in susceptibility towards amphotericin B and 5-fluorocytosine: expression was positively regulated by CnCrz1 and CnYap1 in response to fluconazole	-	Necessary for full virulence of *C. neoformans* in intravenous and in inhalation mouse models; overexpression upon *C. neoformans* phagocytosis; and involved in the resistance against microglia
				*AFR2*	Role in susceptibility towards amphotericin B and 5-fluorocytosine	-	-
				*MDR1*	Confers itraconazole resistance upon hyperexpression in *S. cerevisiae;* role in susceptibility towards amphotericin B and 5-fluorocytosine	-	-
	*Cryptococcus gattii*	23	2	*MDR1*	Confers fluconazole resistance in *S. cerevisiae*	-	Overexpressed in cells recovered from the brain of infected mice
				*PDR11*	Necessary for fluconazole resistance in the VGII clinical strain	-	Overexpressed in cells recovered from the lungs of infected mice

**Table 2:** MFS transporters of *Candida*, *Aspergillus*, and *Cryptococcus* species, focusing their roles in MDR and physiological functions, as well as their pathogenicity and virulence features.

	Species	Total #	Characterised #	Characterised ORFs	Role in MDR	Physiological Role	Pathogenicity and Virulence Features
**MFS Proteins**	*Candida albicans*	26	8	*orf19.5604/MDR1*	Major mediator of azole resistance; methotrexate is preferred substrate; overexpression in drug-resistant clinical isolates confers fluconazole resistance; repressed in young biofilm	-	Necessary for full virulence in *C. albicans* in immunocompetent and immunocompromised mice models
				*orf19.6577/FLU1*	Involved in the resistance towards fluconazole, ketoconazole, and itraconazole; confers fluconazole resistance in *S. cerevisiae*	Involved in histatin-5 efflux	-
				*orf19.2160/NAG4*	Required for wild-type cycloheximide resistance	-	Required for wild-type mouse virulence
				*orf19.2158/NAG3*	Required for wild-type cycloheximide resistance	-	Required for wild-type mouse virulence; Spider biofilm repressed
				*orf19.2151/NAG6*	Required for wild-type cycloheximide resistance	-	Required for wild-type mouse virulence
				*orf19.508/QDR1*	-	Involved in lipid homeostasis	Involved in biofilm architecture and thickness and virulence
				*orf19.6992/QDR2*	-	Involved in lipid homeostasis	Involved in biofilm architecture and thickness and virulence in a murine model of hematogenously disseminated candidiasis
				*orf19.136/QDR3*	-	Involved in lipid homeostasis	Involved in biofilm architecture and thickness and virulence in a murine model of hematogenously disseminated candidiasis
	*Candida glabrata*	15	8	*CAGL0J09944g/AQR1*	Involved in resistance to flucytosine and imidazoles	Involved in resistance to acetic acid	-
				*CAGL0G03927g/TPO1_1*	Putative drug:H^+^ antiporter, involved in efflux of clotrimazole; required for resistance to clotrimazole and other drugs	Involved in the resistance to histatin-5; involved in spermine resistance	Involved in virulence
				*CAGL0E03674g/TPO1_2*	Putative drug:H^+^ antiporter, involved in efflux of clotrimazole; required for resistance to clotrimazole and other drugs	Involved in fatty acid and sterol homeostasis upon biofilm formation; involved in spermine resistance	Involved in virulence in the *G. mellonella* model and biofilm formation
				*CAGL0I10384g/TPO3*	Confers imidazole and triazole drug resistance; activated by CgPdr1	Involved in polyamine homeostasis	-
				*CAGL0G08624g/QDR2*	Confers imidazole drug resistance, involved in clotrimazole efflux; activated by CgPdr1; upregulated in azole-resistant strain	-	-
				*CAGL0H06017g/FLR1*	Confers resistance to benomyl; gene is downregulated in azole-resistant strain	-	-
				*CAGL0H06039g/FLR2*	Multidrug transporter of the major facilitator superfamily involved in 5-flucytosine resistance	-	-
				*CAGL0M06281g/DTR1*	-	Acetate exporter in the plasma membrane	Required for virulence in *G. mellonella* model
	*Candida tropicalis*	26	1	*MDR1*	Overexpression in resistant clinical isolates and upon biofilm formation	-	-
	*Candida parapsilosis*	34	1	*CPAR2_301760/MDR1*	Member of the MDR family of major facilitator transporter superfamily; putative drug transporter; expression increased in fluconazole and voriconazole resistant strains	-	-
	*Candida dubliniensis*	14	1	*Cd36_63890/MDR1*	Predicted multidrug transporter of ABC superfamily, involved in multidrug resistance	-	-
	*Aspergillus fumigatus*	278	6	*Afu6g09710/GLIA*	-	Predicted major facilitator type glioxin transporter, encoded in the putative gliotoxin biosynthetic gene cluster	
				*Afu1g05010/MFS56*	Putative MFS transporter; mutation causes increased azole sensitivity	-	-
				*Afu3g03500/MDR3*	Putative multidrug resistance protein; transcript upregulated in response to amphotericin B; displays itraconazole-increased expression in resistant mutants	-	-
				*Afu8g05710/MFSA*	Highly expressed during voriconazole stress	Putative major facilitator superfamily (MFS) sugar transporter	Calcium induced; transcript upregulated in conidia exposed to neutrophils
				*Afu1g15490/MFSB*	Putative major facilitator superfamily (MFS) transporter; highly expressed during voriconazole stress	-	-
				*Afu1g03200/MFSC*	Putative major facilitator superfamily (MFS) transporter; highly expressed during voriconazole stress	-	-
	*Cryptococcus neoformans*	86	3	*CNA07070*	-	Dityrosine transporter	-
				*CNC03290*	-	-	-
				*CND00440/AFLT*	-	-	-
